# The Hypolipidemic and Anti-Inflammatory Activity of Boronated Aromatic Amino Acids in CF_1_ Male Mice

**DOI:** 10.1155/MBD.1999.337

**Published:** 1999

**Authors:** Merrill C. Miller, A. Sood, Bernard F. Spielvogel, Iris H. Hall

**Affiliations:** 1 Division of Medicinal Chemistry and Natural Products University of North Carolina Chapel Hill North Carolina 27559-7360 USA; 2 Boron Biologicals Inc. Raleigh North Carolina 27606 USA

## Abstract

The boronated aromatic amino acids were shown to be potent hypolipidemic agents in mice lowering both serum cholesterol and triglycerides after 16 days. Selective compounds were as effective as the clinical standards. Furthermore, the compounds were effective anti-inflammatory agents reducing local and central pain as well as suppressing LPS induced endotoxic shock in mice. These agents inhibited lysosomal and proteolytic enzymes of the liver and macrophages as a part of their mechanism of action.


### THE HYPOLIPIDEMIC AND ANTI-INFLAMMATORY ACTIVITY OF BORONATED AROMATIC AMINO ACIDS IN $CF_1$ MALE MICE

Merrill C. Miller, III[1], A. Sood[2], Bernard F. Spielvogel[2], and Iris H. Hall[1]*

[1] Division of Medicinal Chemistry and Natural Products, University of North Carolina, Chapel Hill, North Carolina 27559-7360, USA
[2] Boron Biologicals, Inc., Raleigh, North Carolina, 27606, USA

### ABSTRACT
The boronated aromatic amino acids were shown to be potent hypolipidemic agents in mice lowering both serum cholesterol and triglycerides after 16 days. Selective compounds were as effective as the clinical standards. Furthermore, the compounds were effective anti-inflammatory agents reducing local and central pain as well as suppressing LPS induced endotoxic shock in mice. These agents inhibited lysosomal and proteolytic enzymes of the liver and macrophages as a part of their mechanism of action.

### INTRODUCTION
L-Phenylalanine, tyrosine, proline, histidine and tryptophan boronated derivatives as well as their metal complexes have recently been shown to be potent antineoplastic or cytotoxic agents [1-6]. Previously with the boron analogues of α-amino acids, i.e amine-carboxyboranes, heterocyclic amine boranes and 2'-deoxynucleoside boranes other biological activities, e.g. hypolipidemic [7-13], antiarthritic, antiinflammatory, antiosteoporoeous[14-19] have been demonstrated in rodents. The present investigation involved the examination of boronated aromatic amino acids as potent hypolipidemic or anti-inflammatory agents in mice at 8 mg/kg, I.P.

### MATERIAL AND METHODS
### Source of Compounds
All of the boronated aromatic amino acids or their metal complexes were synthesized and published previously: 2-[(N-methylmethanamine)methyl-carbonyl]amino-3-phenyl-methyl propanoate **3** [6], ], (N,N-dimethylmethanamine)dihydro[[[1-(phenylmethyl)-2-methoxy-2-oxoethyl]amino]carbonyl]boron **4** [3], (N-methylmethanamide)dihydro[[[1-(phenylmethyl)-2-methoxy-2-oxoethyl]amino]carbonyl]boron **5** [3]; (methanamine)dihydro[[[1-phenylmethyl)-2-methylamino-2-oxoethyl]amino]carbonyl]boron **6** [3], sodium –2-(N,N-dimethylmethanamine)dihydroboron-carbonyl]amino-3-phenyl-propanoate **7** [5], tetrakis-{2-[N,N-dimethylmethanamine)dihydroboron-carbonyl]amino-3-phenyl-propyl carboxylate}-bis-{2-[N,N-dimethyl-methanamine)dihydroboron-carbonyl]amino-3-phenyl-propanoate}dicopper(II) **8** [5], (ammonia)dihydro[[[1-(phenylmethyl)-2-methoxy-2-oxoethyl]amino]carbonyl] boron **9** [3], (N,N-dimethyl-methan-amine)dihydro[[[1-(4-hydroxyphenyl)-methyl)-2-methoxy-2-oxoethyl]amino]carbonyl]boron **10** [3], (N-methylmethanamide)dihydro[[1-[(4-hydroxyphenyl)-methyl]-2-methoxy-2-oxoethyl]amino]carbonyl]boron **11** [3], (methanamine)dihydro[[1-[(4-hydroxyphenyl)-methylamino-2-oxoethyl]amino]carbonyl]boron **12** [3], (ammonia)dihydro[[[1-(4-hydroxyphenyl)-methyl)-2-amino-2-oxoethyl]amino]carbonyl]boron **13** [3], (cyano)dihydro[[1-(phenylmethyl)-2-amino-2-oxoethyl]amino]boron **14** [3], N-acetyl-4-boronphenylalanylphenylalanine methyl ester **15** [2], N-acetyl-4-boronphenylalanyltyrosine methyl ester **16** [2], N-[(trimethylamineboryl)-carbonyl]-L-histidinemethyl ester 17 [4], N-[(trimethylamineboryl)-carbonyl]L-tryphophan methyl ester **18** [4]. N-[(trimethylamineboryl)-carbonyl] -L-proline methyl ester **19** [2]. L-phenylalanine **1** [Alrich Chemical Co.], L-phenylalanine methyl ester hydrochloride **2** [Aldrich Chemical, Co.] were purchased commercially.
All substrates, co-factors and standard drugs were purchased from Sigma Chemical Co.

### Pharmacological Methods
*Hypolipidemic Assay.*
$CF_1$ male mice (~28g) were administered drugs prepared in 1% carboxymethylcellulose [CMC] by homogenization at 8 mg/kg/day, I.P. for 16 days. On days 9 and 16 the mice were bled from the tail vein collected in capillary tubes which were centrifuged at 3000 x g for 3 min. Total cholesterol was determined on the serum using the Libermann-Burchard method [20] determined at 620 nm. Serum triglycerides were determined on day 16 using commerical kits from Sigma Chemical, Co. read at 580 nm. Clofibrate was determined at 150 mg/kg/day I.P. and lovastatin was determined at 8 mg/kg/day I.P., which are the drugs' standard therapeutic doses.

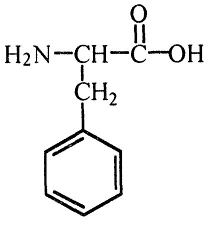

**1**

L-Phenylalanine

1-(phenylmethyl)-2-hydroxy-2-ethyl amine

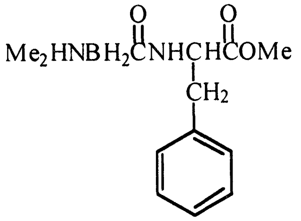

**5**
(N-methylmethanamine)dihydro[[[1-(phenylmethyl)-2-methoxy-2-oxoethyl]amino]carbonyl]boron

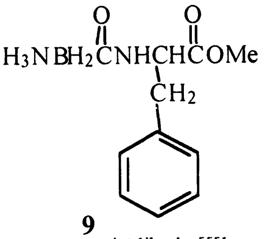

**9**
(Ammonia)dihydro[[[1-(phenylmethyl)-2-methoxy-2-oxoethyl]amino]carbonyl]boron

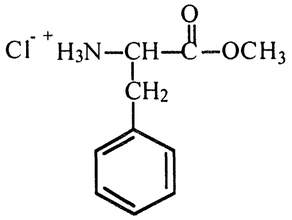

**2**
1-(Phenylmethyl)-2-methoxy-2-oxyethyl-amine hydrochloride

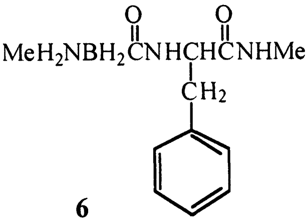

**6**
(Methanamine)dihydro[[[1-(phenylmethyl)-2-methylamino-2-oxoethyl]amino]carbonyl]boron

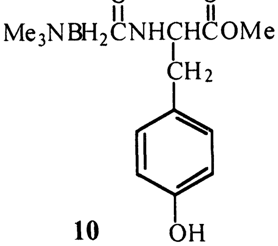

**10**
(N,N-dimethylmethanamine) dihydro[[[1-[(4-hydroxyphenyl)-methyl]-2-methoxy-2-oxoethyl]amino]carbonyl]boron

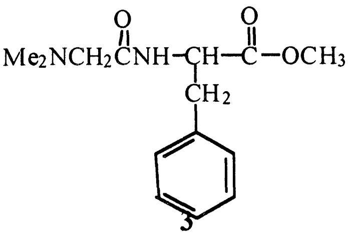

**3**
2-[(N-Methylmethanamine) methyl-carbonyl]amino-3-phenyl-methyl- propanoate

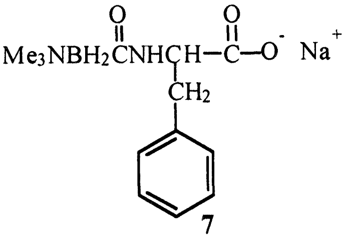

**7**
Sodium-2-{N,N-dimethyl-methanamine) dihydroboron-carbonyl]amino-3-phenyl-propanoate

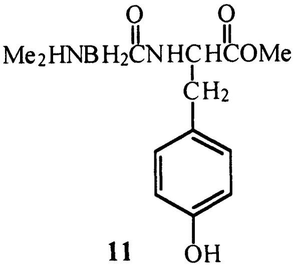

**11**
(N-methylmethanamine) dihydro[[[1-[(4-hydroxyphenyl)-methyl]-2-methoxy-2-oxoethyl]amino]carbonyl]boron

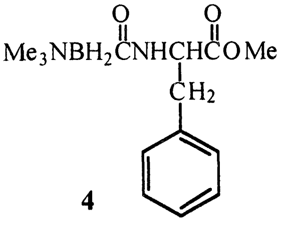

**4**
(N,N-dimethylmethanamine)dihydro[[[1-(phenylmethyl)-2-methoxy-2-oxoethyl]amino]carbonyl]boron

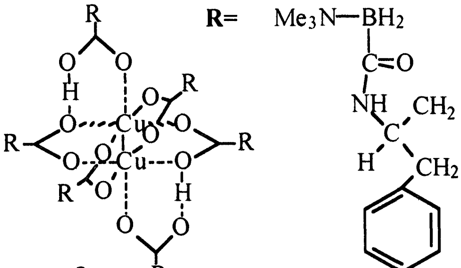

**8**
Tetrakis--{2-[N,N-dimethyl-methanamine) dihydroboron-carbonyl]amino-3-phenyl-propyl-carboxylato)-bis-(2-[N,N-dimethyl-methanamine) dihydroboron-carbonyl]amino-3-phenyl-propanoate) dicopper (II)

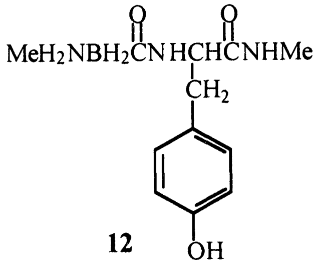

**12**
(Methanamine)dihydro[[[1-[(4-hydroxyphenyl)-methyl]-2-methylamino-2-oxoethyl]amino]carbonyl]boron

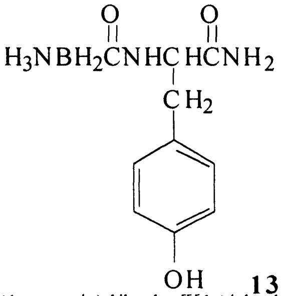

**13**

(Ammonia)dihydro[[[1-(4-hydroxyphenyl)-methyl]-2-amino-2-oxoethyl]amino]carbonyl]boron

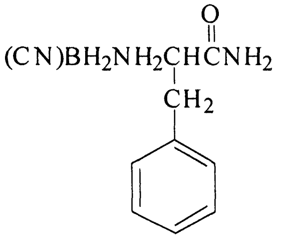

**14**

(Cyano)dihydro[[1-(phenylmethyl)-2-amino-2-oxoethyl]amino]boron

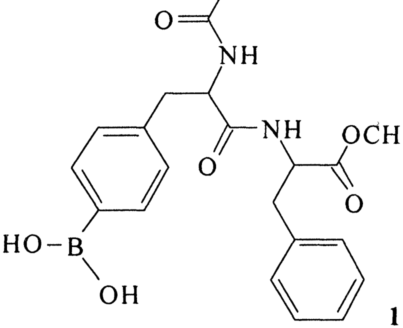

**15**

N-acetyl-4-boronophenylalanylphenylalanine methyl ester

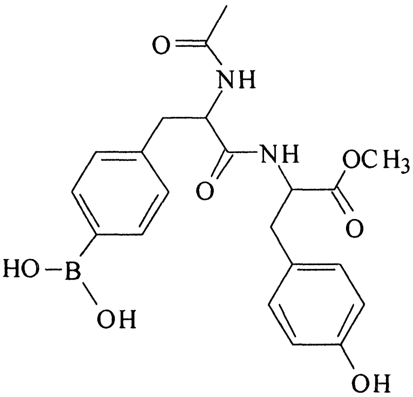

**16**

N-acetyl-4-boronophenylalanyltyrosine methyl ester

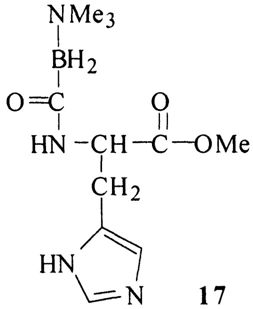

**17**

N-[(Trimethylamine-boryl)-carbonyl]-L-histidine methyl ester

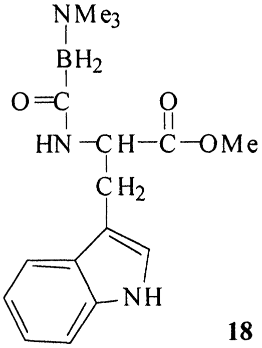

**18**

N-[(Trimethylamine-boryl)-carbonyl]-L-tryptophan methyl ester

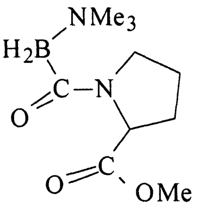

**19**

N-[(trimethylamine-boryl)-carbonyl]-L-proline methyl ester

Figure 1 Structures of Aromatic Boronated Amino Acids

*Anti-inflammatory Activity.*
$CF_1$ male mice (~25 g) were administered drugs at 8 mg/kg in 1% CMC I.P, at 3 h and again 30 min prior to the injection in 0.2 ml of carrageenan in 0.9% saline into the plantar surface of the high hind foot. Saline was injected into the left hind foot which served as the standard base line. After 3 h both feet were excised at the tibiolarsal (ankle) joint according to the modified method of Winter et al.[21], resulting in 84 mg increase in the paw weight of the control mice.
*Local Analgesic Activity-Writhing Reflex.*
Male $CF_1$ male mice (~25g) were administered test drugs at 8 mg/kg I.P. 20 min prior to the administration of 0.5 ml of 0.6% acetic acid, I.P. [22]. After 5 min the number of stretches, characterized by repeated contractions of the abdominal musculature accompanied by extension of the hind limbs for the next 10 min were counted. The control mice afforded 61 stretch reflexes in 10 min.
*Hot Plate Tail Flick Activity -Central Analgesic Activity.*
$CF_1$ male mice (~28g) were administered drugs at 8 mg/kg, I.P., 15 min prior to placement on a hot plate maintained at 100 °C. The time elapse for the tail to be raised from the surface of the hot plate was determined using a digital read-out connected to the hot plate [23]. The tail flick response of the control mice was 12.12 sec.
*Endotoxic Shock Protection.*
$CF_1$ male mice (~25g) were administered *Salmonella* lipopolysaccharide (LPS) at 10 mg/kg, IP which is a dose that is lethal by 100% within 48-52 h [24] . Drugs were administered at 8 mg/kg, 2 hr before and 2 hr after the injection of LPS and every 24 h there after up to 48 h. Deaths were recorded daily for the length of the study. The percentage deaths at 52 h was calculated and compared to the control value that afforded 16% survival of the animals at that time.

**Table 1 The Hypolipidemic Effects of Boronated Aromatic Amino Acids at 8 mg/kg I.P**
Percent of Control (mean $\pm$ standard deviation)

| N =6 | Serum Cholesterol Day 9 | Serum Cholesterol Day 16 | Serum Triglycerides Day 16 |
|---|---|---|---|
| Control 1% CMC | $100\pm5^a$ | $100\pm6^b$ | $100\pm4^c$ |
| **1** | $90\pm5$ | $90\pm6$ | $94\pm6$ |
| **2** | $69\pm5$* | $61\pm5$* | $63\pm5$* |
| **3** | $76\pm4$* | $74\pm5$* | $80\pm5$* |
| **4** | $75\pm6$* | $52\pm3$* | $67\pm5$* |
| **5** | $86\pm5$ | $69\pm5$* | $92\pm6$ |
| **6** | $75\pm4$* | $52\pm3$* | $92\pm5$ |
| **7** | $71\pm5$* | $68\pm4$* | $88\pm5$ |
| **8** | $72\pm5$* | $63\pm4$* | $61\pm5$* |
| **9** | $87\pm6$ | $71\pm5$* | $98\pm5$ |
| **10** | $72\pm5$* | $68\pm6$* | $55\pm5$* |
| **11** | $73\pm4$* | $67\pm5$* | $68\pm6$* |
| **12** | $73\pm5$* | $74\pm4$* | $83\pm5$ |
| **13** | $82\pm6$ | $68\pm5$* | $96\pm6$ |
| **14** | $64\pm3$* | $55\pm4$* | $77\pm4$* |
| **15** | $68\pm5$* | $62\pm4$* | $73\pm4$* |
| **16** | $66\pm4$* | $65\pm6$* | $78\pm5$* |
| **17** | $71\pm7$* | $69\pm5$* | $65\pm6$* |
| **18** | $75\pm3$* | $68\pm6$* | $82\pm6$ |
| **19** | $65\pm4$* | $64\pm5$* | $82\pm5$ |
| **Lovastatin 8 mg/kg** | $85\pm4$ | $82\pm5$ | $86\pm7$ |
| **Clofibrate 150 mg/kg** | $88\pm4$ | $78\pm5$ | $75\pm5$* |

a = 125 mg/dL; b = 127 mg/dL; c = 137 mg/dL.

### Enzyme Assays.

$CF_1$ livers were homogenized in 0.25 M sucrose + 0.001 M EDTA, pH 7.2. Mouse macrophages J774A were maintained in Dulbecco's modified medium (DMEM) + 15% fetal calf serum + P/S were homogenized.

All of the methods for the enzyme studies have been described previously [16]. Acid phosphatase, elastase, trypsin and cathepsin activities were determined after 30 min incubation of drugs at $10^{-8}$ M prepared in 1% CMC at 37°C. Acid phosphatase activity was determined using 0.1 M β–glycerolphosphate in 0.1 M acetate buffer, pH 5.0. The reaction was stopped with 10% TCA and centrifuged at 3000 x g x 6 min. The supernatant inorganic phosphate was determined by the spectrophotometric method. The net inorganic phosphate released from 30 min was corrected by subtracting the blank. Cathepsin activity was determined using 2% azocasein as the substrate in 0.1 M acetate buffer, pH 5.0 and the hydrolyzed acid-soluble peptide fragment was analyzed at 366 nm and corrected for the blank. Trypsin proteolytic activity was determined by the method of Schleuning and Fritz [25] using 6 mM N-benzoly-L-arginine ethyl ester (BAEE) in 0.1 M Tris buffer, pH 8.0. The hydrolyzed product was determined at 253 nm and the blank subtracted. Elastase activity was determined by the method of Kleinerman et al. [26] with 2 units of porcine pancreatic elastase (Sigma, Type III), N-succinyl-L-alanyl-L-alanine-p-nitroanilide (Sigma, 100 mg in 5 ml methyl-2-pyrrolidone) in 0.2 M Tris buffer, pH 8.0. The cleaved product *p*-nitroanilide was determined at 410 nm and the blank substracted.

Table 2 **The Anti-inflammatory Effects of Boronated Aromatic Amino Acids at 8 mg/kg I.P**

Percent of Control (mean ± standard deviation) N=6

| | Anti -inflammatory | Writhing | Hot Plate -Tail Flick | LPS ≈ -Protection |
|---|---|---|---|---|
| Control | 100±5 | 100+4 | 100 | 16 |
| 1 | 83±5 | 77±5* | 107 | 17 |
| 2 | 90±4 | 50±4* | | 83 |
| 3 | 68±7* | 48±3* | 113 | 100 |
| 4 | 68±5* | 48±5 | 139 | 33 |
| 5 | 60±5* | 15±3* | 85 | 100 |
| 6 | 77±5* | 83±4 | | 100 |
| 7 | 60±6* | 97±5 | 83 | 83 |
| 8 | 107±6 | 97±6 | 120 | 67 |
| 9 | 80±5 | 16+2* | | 83 |
| 10 | 63±4* | 25+4* | 148 | 83 |
| 11 | 70±5* | 19±3* | 218 | 100 |
| 12 | 72±4* | 21±4* | 131 | 100 |
| 13 | 68±3* | 15±3* | 189 | 83 |
| 14 | 59±4* | 42±4* | 96 | 83 |
| 15 | 76±4* | | | |
| 16 | 75±4* | | | |
| 17 | 70±7* | 104±6 | | 67 |
| 18 | 94+7 | 94±4 | | 83 |
| 19 | 70+4+8 | 83±4 | | |
| Standards | | | | |
| Indomethacin 8 mg/kg | 74+4* | 43±4* | | 50 |
| Phenyl-butazone 50 mg/kg | 53±4* | | | |
| Morphine 1mg/kg | --- | | 213 | |
| Dexamethasone 1mg/kg | --- | | | 67 |
| Pentoxifylline 50 mg/kg | --- | | | 67 |

The control values for these assays are located in the Methods section.

### RESULTS

The boronated amino acids and their metal complexes demonstrated significant hypolipidemic effects in mice at 8 mg/kg I.P. [Table 1]. Compounds **6** and **14** caused greater than 40% reduction of total serum cholesterol levels after 16 days. Compounds **2, 4, 5, 7, 8, 10, 11, 13** and **15-19** afforded greater than 30% reduction of serum cholesterol after 16 days which was significantly better than the standards clofibrate and lovastatin at their therapeutic doses. Serum triglycerides were reduced 45% by compound **10**. Compounds **2, 6, 8, 11** and

**17** lowered serum triglyceride levels greater than 30% which was greater than the standards' effects. L-Phenylalanine itself was not active in either assay. The addition of the methyl ester did result in an active compound indicating that the boron atom was not necessarily important for hypolipidemic activity in mice.

The boronated aromatic amino acid demonstrated anti-inflammatory activity in mice [Table 2] with compounds **3-5**, **7**, **10**, **13**, **14** and phenylbutazone demonstrating greater than 30% inhibition of edema. Compounds **6**, **11**, **12**, **15**, **16**, **17**, **19** and indomethacin caused greater than 20% reduction. Compounds **2-5**, **9-14** and indomethacin afforded at least 50% inhibition of the local analgesic writhing reflex. Central analgesic activity similar to that demonstrated by morphine at 1 mg/kg with an increase of 113% above the control value was observed for compounds **11** and **13** which demonstrated increases of 118 % and 89%, respectively at 8 mg/kg. Compounds **4**, **8**, **10** and **12** demonstrated a significant increase but were not as potent as morphine. Protection of 100% against LPS induced endotoxin induced shock was afforded by compounds **3**, **5**, **6**, **11** and **12** at 8 mg/kg. Compounds **2**, **7**, **9**, **10**, **13**, **14** and **18** caused 83% protection which was better than compound **8** and **17** at 8 mg/kg and dexamethasone at 1 mg/kg and pentoxifylline at 50 mg/kg which caused 67% protection in mice.

The boronated aromatic amino acids *in vitro* demonstrated the ability to suppress the activities of proteolyic and lysosomal hydrolytic enzymes in $CF_1$ mouse liver homogenates and cultured mouse J774A macrophages [Table 3]. In mouse liver compounds **3-5**, **7**, **12-17** and **19** afforded greater than 40% inhibition of elastase activity. Liver cathepsin activity was reduced greater than 30% by compounds **4**, **5**, **7**, **9** **13**, **17** and **19**. Liver trypsin proteolytic activity was suppressed 94% by compound **7**, but compounds **11-17** caused greater than 50% reduction of activity. In the cultured mouse macrophages elastase activity was reduced at least 30% by compounds **3**, **5**, **8** **10**, **12**, **15** and **17**. Macrophage cathepsin activity was inhibited greater than 35% by compounds **5**, **11**, **12**, **14**, **16**, **17**, and **19**. Trypsin activity was reduced at least 30% by compounds **10** and **16**. Macrophage acid phosphatase activity was reduced 40% by compound **5** and 30% by compound **13**.

Table 3 The Effects of Boronated Aromatic Amino Acid on Lysosomal Enzyme Activities
Percent of Control (Standard Deviations)

| | -----$CF_1$ Mouse Liver--------------- | | | ---------- J774A Macrophages----------------------- | | | |
|---|---|---|---|---|---|---|---|
| N =4 | Elastase | Cathepsin | Trypsin | Elastase | Cathepsin | Trypsin | Acid phosphatase |
| **Control** | 100±5 | 100±4 | 100±7 | 100±5 | 100±6 | 100±4 | 100±5 |
| **1** | 99±4 | 96±5 | 97±4 | 103±5 | 98±5 | 102±6 | 97±7 |
| **2** | 78±5* | 86±6 | 87±5 | 73±4* | 84±5 | 92±5 | 81±4* |
| **3** | 53±3* | 75±4* | 135±7* | 67±4* | 89±6 | 74±5* | 76±4* |
| **4** | 43±4* | 65±5* | 79±4* | 73±4* | 113±5 | 76±5* | 93±6 |
| **5** | 48±3* | 66±3* | 142±5* | 68±3* | 54±3* | 82±5 | 60±4* |
| **6** | 74±4* | 75±3* | 95±5 | 82±6 | 74±4* | 80±3* | 88±6 |
| **7** | 51±4* | 60±4* | 6±2* | 72±5* | 93±5 | 94±6 | 97±5 |
| **8** | 68±4* | 73±5* | 81±4* | 69±4* | 71±5* | 81±5* | 91±4 |
| **9** | 73±4* | 67±3* | 74±4* | 78±5* | 79±4* | 86±4 | 99±6 |
| **10** | 65±5* | 72±4* | 117±6 | 67±6* | 89±6 | 68±5* | 95±5 |
| **11** | 60±4* | 85±5 | 30±3* | 82±5 | 57±5* | 88±5 | 85±5 |
| **12** | 59±4* | 91±6 | 41±4* | 63±4* | 61±5* | 90±6 | 104±7 |
| **13** | 49±3* | 66±5* | 31±3* | 70±5* | 72±4* | 104±5 | 70±4* |
| **14** | 54±4* | 88±4 | 38±3* | 76±5* | 51±4* | 76±5* | 91±6 |
| **15** | 55±6* | 75±5* | 36±2* | 69±6* | 94±6 | 84±6 | 103±5 |
| **16** | 57±4* | 74±3* | 44±4* | 82±7 | 57±4* | 57±4* | 94±4 |
| **17** | 54±3* | 68±3* | 44±5* | 65±6* | 62±5* | 81±4* | 108±5 |
| **18** | 63±7* | 71±4* | 62±4* | 76±5* | 66±4* | 71±3* | 90±5 |
| **19** | 58±5* | 68±4* | 81±5 | 96±6 | 57±4* | 96±5 | 85±4 |

### DISCUSSION

The boronated aromatic peptides demonstrated good hypolipidemic activity that was consistent with other boronated compounds. They perhaps were more effective in lowering serum cholesterol levels after 16 days than lowering serum triglycerides levels. (N,N-dimethylmethanamine)dihydro[[[1-(phenylmethyl)-2-methoxy-

2-oxoethyl]amino]carbonyl]boron **4** and (cyano)dihydro[[1-(phenylmethyl)-2-amino-2-oxoethyl]amino]boron **14** appear to be the best compounds in this respect and they were markedly more
potent at 8 mg/kg than the standards clofibrate at 150 mg/kg and lovastatin at 8 mg/kg. There did not appear to significant differences between the phenylalanine, tyrosine, proline, histidine and tryptophan derivatives and their effects on lowering cholesterol levels. (N,N-dimethymethanamine)dihydro[[[1-(4-hydroxyphenyl)methyl)-2-methoxy-2-oxoethyl]amino]carbonyl]boron **10** was the most potent agent in lowering serum triglyceride levels on day 16. Again this magnitude of reduction of triglycerides was highly significant compared to the standard drug effects on this lipid component.
Their anti-inflammatory effects of the boronated aromatic amino acids was not as significant as those observed for the α–amino boranes [16]. The sodium-2-(N,N-dimethylmethanamine)dihydroboron carbonyl)amino-3-phenylpropanoate **7** (cyano)dihydro[[1-(phenylmethyl)-2-amino-2-oxoethyl]aminoboron **14** achieved the same degree of reduction of induced edema as the standard phenylbutazone at 50 mg/kg x 2. The agents were better in reducing local pain [writhing] with (methanamine)dihydro[[[1-(phenylmethyl)-2-methylamino-2-oxoethyl]amino]carbonyl]boron **5** as well as the tyrosine derivatives **9-13** affording better activity than the standard indomethacin. The other boronated aromatic amino acids were not significantly active in reducing local pain. (N-methylmethanamide)dihydro[[[1-(4-hydroxyphenylmethyl)-2-methoxy-2-oxoethyl ]amino]carbonyl]boron **11** was the only compound that was as active as morphine in reducing central pain although compound **13** was significantly effective. The phenylalanine derivatives did not demonstrate any ability to block central pain. The boronated aromatic acids did protect the mouse from LPS induced shock and many of the tested derivatives were more potent than the standards dexamethasone and pentoxifylline at their therapeutic doses. Those compounds without a boron atom, compounds **1** and **2** offered no protection against LPS induced shock. Previously amine-carboxyboranes were shown to inhibit proteolytic and lysosomal hydrolytic enzyme activities [16]. The present study would suggest that similar mechanisms of action are caused by the boronated aromatic amino acids for their anti-inflammatory activity. The amine-carboxyboranes also suppressed prostaglandin synthetase and cytokine, e.g. Il-1 and TNFα release as well as the migration of white cells to the inflammation site [16]. Whereas further investigation of this group of compounds is required, they may offer some therapeutic use in the future because of the low dose required for their *in vivo* activity as hypolipidemic and anti-inflammatory agents.

Refereences

1. C.K. Sood, A. Sood, B.F. Spielvogel and I.H. Hall, *Eur. J. Med. Chem.* 1990, **25**, 301-308.
2. I.H. Hall, E.S. Hall, M.C. Miller, III, A. Sood and B.F. Spielvogel, *Amino Acids* 1993, **4** , 287-302.
3. S. Karthikeyan, A.Sood, J. Tomasz, B.F. Spielvogel and I.H. Hall, *Amino Acids* 1995, **8**, 323-335.
4. M.C. Miller, A. Sood, B.F. Spielvogel and I.H. Hall, *Anticancer Res.* 1997, **17**, 3299-3306.
5. M.C. Miller, A. Sood, B.F. Spielvogel and I.H. Hall, *Met.Based Drugs*, **5**, 1-9, 1997.
6. M.C. Miller, III< A. Sood, B.F. Spielvogel, R.P. Shrewsbury and I.H.Hall, *Met.Based Drugs* 1996, **3**, 219-226.
7. I.H. Hall, B. F. Spielvogel, A.T. McPhail and W.L. Williams, Jr. *J. Pharm Sci* 1984, **73**, 973-977.
8. I.H. Hall, B. F. Spielvogel, T. S. Docks and B.J. Brotheron, *Res. Commun. Chem. Pathol, Pharmocol.* 1989, **65**, 297-317.
9. I.H. Hall, A. Sood and B.F. Spielvogel, *Biomed. Pharmther.* 1991, **45**, 333-341.
10. I.H. Hall, B.S. Burnham, B.R. Shaw, K.G. Rajendran, A. Sood and B.F. Spielvogel, *Biomed. Pharmther.* 1993, **47**, 79-87.
11. I.H.Hall, S. Y. Chen,, K.G. Rajendran , A. Sood and B.F. Spielvogel, *Envir. Health Persp.* 1994, **102**, Suppl. 3, 211-214.
12. I.H. Hall, D.J. Reynolds, O.T. Wong, A. Sood and B.F. Spielvogel, *Met. Based Drugs* 1995, **2**, 65-72.
13. B.S. Burnham, A. Sood, B.F. Spielvogel, S.Y. Chen and I.H. Hall, *Met. Based Drugs* 1996, **3**, 173-183.
14. I.H. Hall, K. G. Rajendran, S.Y. Chen, V.M. Norwood III, K.W. Morse, A. Sood, and B.F. Spielvogel, *Appl. Oranomet. Chem.* 1994, **8**, 473-480.
15. K.G. Rajendran, B.S. Burnham, S.Y. Chen, A. Sood, B.F. Spielvogel, B.R. Shaw and I.H. Hall, *J. Pharm. Sci.* 1994, **83**, 1391-1395.
16. I. H. Hall, K.G. Rahendran, S.Y. Chen, O.T. Wong, A. Sood, and B.F. Spielvogel, *Arch. Pharm.* 1995, **328**, 39-44.
17. K.G. Rajendran, A. Sood, B.F. Spielvogel, I.H. Hall, V.M. Norwood, III and K.W. Morse, *Appl. Organomet. Chem.* 1995, **9**, 111-119.

18. I.H. Hall, B.S. Burnham, S.Y. Chen, A. Sood, B.F. Spielvogel and K. W. Morse, *Met. Based Drugs* 1995, **2**, 1-12.
19. K.G. Rajendran, S.Y. Chen, A. Sood, B.F. Spielvogel and I.H. Hall, *Biomed. Pharmther.* 1995, **49**, 131-140.
20. A.T. Ness, J.V. Pastewka and A.C.Peacock, *Clin. Chem. Acta* 1964, **10**, 229-237.
21. C.A. Winter, E. Risley and G. Nuss, *Proc. Soc. Expt. Biol. Med.* 1992, **111**, 544-547.
22. A.P. Roszkowski, W.H. Rooks, A. Tomonlonis and L.M. Miller, *J. Pharm. Exp. Ther.* 1971, **179**, 114-123.
23. I.H. Hall, J.E. Hall, M. Mohseni, Z. Sajadi, *J. Pharm. Sci.* 1980, **69**, 1451- 1452.
24. L. Noronha-Blob, V.C. Lowe, M. Weitzberg, R.M. Burch, *Eur. J. Pharm.* 1991, 387-388.
25. W.D. Scleuning, H.Fitz, *Meth. Enzymolog.* 1976, 1117-1139.
26.J. Kleinerman, V. Ranga, J. Rynbrant, J. Sorensen, J. Powers, *Am.Rev.Respir. Dis.* 1980, **121**,381-387.

**Received: June 29, 1999 - Accepted: August 24, 1999 -
Received in revised camera-ready format: August 25, 1999**

